# Engineering food crops to grow in harsh environments

**DOI:** 10.12688/f1000research.6538.1

**Published:** 2015-09-02

**Authors:** Damar López-Arredondo, Sandra Isabel González-Morales, Elohim Bello-Bello, Gerardo Alejo-Jacuinde, Luis Herrera

**Affiliations:** 1 StelaGenomics, Irapuato, Guanajuato, 36821, Mexico; 2 Laboratorio Nacional de Genómica para la Biodiversidad, Centro de Investigación y de Estudios Avanzados del Instituto Politécnico Nacional, Irapuato, Guanajuato, 36821, Mexico

**Keywords:** plant nutrition, abiotic stress, plant development, grain yield, gene overexpression, bacterial genes, biotechnology

## Abstract

Achieving sustainable agriculture and producing enough food for the increasing global population will require effective strategies to cope with harsh environments such as water and nutrient stress, high temperatures and compacted soils with high impedance that drastically reduce crop yield. Recent advances in the understanding of the molecular, cellular and epigenetic mechanisms that orchestrate plant responses to abiotic stress will serve as the platform to engineer improved crop plants with better designed root system architecture and optimized metabolism to enhance water and nutrients uptake and use efficiency and/or soil penetration. In this review we discuss such advances and how the generated knowledge could be used to integrate effective strategies to engineer crops by gene transfer or genome editing technologies.

## Introduction

Current agricultural systems use numerous crop varieties that have been improved through traditional breeding, which has produced a substantial increase in the yields of many crops, particularly cereals ^1^. However, high input agriculture has been conducted with the excessive use of agrochemicals, including phosphorus (P)- and nitrogen (N)-fertilizers, herbicides, and insecticides. Moreover, the harsh conditions that crops face, such as drought, soil elemental toxicities, extreme temperatures, and high soil impedance, merit special attention because they drastically limit crop yields worldwide ^2, 3^. The predicted increase in the global population to over 9 billion by 2050 ^4^ poses a critical challenge: how to develop effective stress-resistant/tolerant crops that are more competitive and can grow in marginal soils to ensure food production.

The development of efficient gene transfer systems, together with combined “omics” platforms (e.g. genomics, transcriptomics, proteomics and metabolomics), has facilitated the understanding of the physiology and biochemistry of plant adaptive responses to unfavorable environmental conditions and the identification of the key molecular players that control these responses. Several genes encoding transcription factors (TFs), transporters, and metabolic enzymes with a clear potential to improve crops have been identified. Agricultural schemes that use transgenic crops have proven to be effective and complementary alternatives because they provide multiple benefits for farmers (e.g. 37% less pesticide used, 22% higher yields, and 68% more profits); such crops are cultivated today on more than 180 million hectares globally ^5^. The transgenic crops that are currently on the market address the crop yield per unit area by controlling insect attacks and weed competition; however, new transgenic crops that overcome the limitations caused by harsh environments are also starting to be deregulated for commercial use ^5^. In this review, we highlight some relevant transgenic approaches regarding nutrient use efficiency, abiotic stresses and soil physical degradation. These approaches have the potential to increase crop yields in marginal lands with poor soil fertility or low water availability and to expand cropping land into places in which the agro-climatic conditions are favourable but abiotic stress reduces yields and thereby discourages agricultural production.

## The two most limiting nutrients for crop productivity: phosphorus and nitrogen

Among all of the nutrients required by plants, P and N are the most limiting factors for agricultural production in most soils; thus, large amounts of fertilizers are commonly applied to ensure high yields. Although plants are able to use different organic compounds as sources of nutrients, P can be assimilated only in the form of orthophosphate (H _2_PO _4_^-^/HPO _4_^-2^, Pi), whereas N is predominantly taken up as nitrate (NO _3_^-^, Ni) or ammonia (NH _4_^+^) ^6, 7^. Moreover, the availability of Pi in the soil solution is drastically affected by the biogeochemical properties of the soil, making P-fertilization efficiency highly variable and more dependent on external inputs. To ensure high yields, farmers usually apply excessive amounts of both P- and N-fertilizers. This practice is unsustainable because crops use only 20–40% of the applied nutrients; the remainder contributes to environmental pollution, toxic algal blooms, and global warming ^8^. Whereas N-fertilizers are synthesized from atmospheric N through a process that consumes at least 1% of global energy usage, P-fertilizers are produced from phosphate rock, a finite, non-renewable mineral resource. Consequently, both fertilizer and food prices will increase continuously. Therefore, searching for integrated strategies to increase P, N, and water use efficiency is an issue of food security and sustainability for all nations. The following paragraphs discuss the most relevant advances in engineering improved P and N uptake and use efficiency.

### Manipulating key elements of phosphorus and nitrogen metabolism

How can we improve P and N uptake and/or use efficiency in crops? There is no simple answer. This issue is being addressed by attempting to identify the key genes that control the global adaptive responses that plants display to low availability of N and P and to investigate the possible contributions of these genes to enhancing nutrient uptake and use efficiencies. This set of responses includes profound morphological, physiological and metabolic changes, which rely on the induction and repression of numerous genes and allow plants to survive and reproduce under nutrient-deprived conditions ^9– 12^. For instance, under limited-P regimens, plants optimize P use by activating metabolic pathways that require smaller amounts of P-containing compounds, reducing shoot growth and promoting root branching to enhance soil exploration ^10– 12^.

The uptake of N and Pi from the soil is critical and requires specialized transporter proteins ^6, 13– 18^; therefore, overexpression of these transporters has been considered as a potential approach for plant improvement. However, overexpressing Pi transporters has either had little effect on Pi uptake or, in some cases, resulted in toxicity symptoms due to an excessive accumulation of Pi in the shoots ^19, 20^. Interestingly, overexpression of the Phosphate Transporter Traffic Facilitator 1 (PHF1) in rice, responsible for regulating the localization of low- and high-affinity Pi transporters to the plasma membrane ^21^, results in enhanced low-Pi tolerance. Field data demonstrate that grain yield of PHF1-overexpressing plants in a low-Pi soil is higher than that of wild-type (WT) plants, suggesting that post-transcriptional regulation of Pi transporters could also be considered to improve crop performance in soils with low-Pi availability ^22^.

The generation of transgenic plants to improve the N use efficiency has also been attempted in a variety of crop plants by manipulating the flux-limiting enzymes involved in N assimilation ^23– 25^. However, except in the case of alanine aminotransferase (AlaAT) ^26^, as described below, the overexpression of enzymes has not provided reproducible or robust results to indicate that it could be an effective strategy for improving the efficiency of N use.

**Figure 1.**
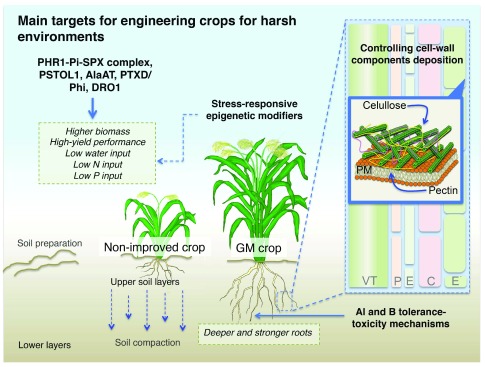
Main targets for engineering crops for harsh environments Engineering DRO1, AlaAT, PSTOL1, PTXD/Phi and the PHR1-Pi-SPX complex represent interesting approaches with the potential to improve crops for harsh environments. In addition, the identification and manipulation of genes involved in cell-wall components synthesis and stress-responsive epigenetic modifiers has great potential for developing optimal root systems and the improvement of plant responses to diverse stimuli. The simultaneous manipulation of some of these elements could bring robust effects to develop crops with high-yield performance, with a consequent decrease in P- and N-fertilizers input. C, cortex; E, endodermis; E´, epidermis; P, pericycle; VT, vascular tissue.

In addition to transporters and key enzymes, some TFs that play crucial roles as master regulators of P and N metabolism have been identified. PHR1 is a member of the MYB transcription family that activates the expression of a large set of the Pi-responsive genes that participate in the low-Pi rescue responses in *Arabidopsis*, and it is evolutionarily conserved from algae to vascular plants ^27^. Overexpressing Phosphate Starvation Response 1 (PHR1 *)* and other TFs, such as Phosphate Starvation-Induced Transcription Factor 1 (PTF1) and OsMYB2P-1, in a variety of crops, such as wheat ^28^, rice ^29^, and maize ^30^, appear to confer low-Pi tolerance and improved grain yield in greenhouse or field trials. Field-testing in different geographical locations and different soil types is required to confirm that the overexpression of these TFs is a robust strategy for improving plant performance under Pi-limiting conditions without affecting performance under optimal Pi availability. Recently, it was reported that in *Arabidopsis* and rice, SPX1 and SPX2 repress the activity of PHR1 as a transcriptional activator in a Pi-dependent manner ^31, 32^. The data published in these reports strongly suggest that the PHR1-SPX1/SPX2 complex is one of the main sensors that regulate the plant response to low-Pi availability. Although no structure of the PHR1-Pi-SPX complex is available, regulation of the interaction between these proteins could become an important target for engineering plants with modulated responses to low-Pi availability. It could be possible to alter PHR1 or SPX1/SPX2 in such a way that the low-Pi response could be modulated to activate processes that enhance Pi uptake and assimilation while preventing the drastic reduction in shoot growth that is generally observed in Pi-starved plants ( Figure 1).

In the case of N, overexpression of the TaNFYA-B1 TF in wheat improved the yield under different regimes of P and N inputs under field conditions ^33^. These results were attributed to enhanced root growth and the up-regulation of N- and Pi-transporters. In addition, the TEOSINTE BRANCHED1/CYCLOIDEA/PROLIFERATING CELL FACTOR1-20 (TCP20) TF was recently identified as a key element in the systemic signaling pathway that directs N foraging in *Arabidopsis* roots, thus opening up the possibility of controlling root plasticity to improve soil exploration capacity in crops ^34^.

Overexpression of TF, transporters or enzymes generally used the CaMV35S promoter, which confers constitutive high expression levels, independently of nutrient availability in the soil, as well as in cells that normally do not express the overexpressed gene and lack the expression of other genes required for efficient nutrient assimilation. Therefore, these approaches must consider cell-specific expression and/or modulation of inter-connected biochemical or regulatory pathways to ensure an appropriate phenotype. Recently, enhancer elements that regulate the transcriptional activation of Pi-starvation responsive genes were reported ^35^. These enhancer elements could be used to design synthetic promoters that could direct high levels of expression while maintaining cell specificity and responsiveness to Pi- or N-deprivation. An additional phase for the improvement of nutrient uptake and use efficiency will be the understanding of the regulatory networks that orchestrate plant responses to nutrient deficiency. The integration of this knowledge will serve to design strategies to direct the enhanced expression of two or more TFs simultaneously, leading to more robust improvements in the key traits to achieve a more sustainable agriculture ( Figure 1). Altering expression of several TFs to have a higher level of induction upon the stress stimuli, or higher cell-specific constitutive expression, could be feasible by introducing enhancer elements by genome editing using the CRISPR/Cas9 system that allows the simultaneous modification of several genes at the same time ^36^.

Interestingly, TF-overexpressing plants that showed an increased yield under N- or Pi-limiting conditions generally developed a more robust root system. This finding corroborates the importance of the root system architecture in soil exploration and nutrient uptake. Therefore, the identification and molecular characterization of quantitative trait loci (QTL) and marker-assisted backcrossing of genes that regulate root traits that improve nutrient uptake and use efficiencies into modern varieties is of the highest importance ^37– 40^. Genes that are responsible for these QTL as well as superior allelic variations in candidate genes, identified in GeneBank collections for instance, could provide powerful potential tools for engineering crops for higher nutrient uptake efficiency in the same or other species by gene transfer or genome editing technologies.

Improvements in P and N metabolism should come from enhanced nutrient uptake and assimilation and/or their subsequent remobilization to support seed or fruit production. Among reported efforts to improve Pi and N use efficiency, we identify the following three promising strategies to develop improved crops, which could make a real contribution to sustainable agriculture: the use of AlaAT to enhance N assimilation ^26^, the use of PHOSPHORUS-STARVATION TOLERANCE 1 (PSTOL1) to enhance P assimilation ^41^, and the development of a novel fertilization system based on the production of transgenic plants that are able to use phosphite (Phi) instead of Pi as a P source ^42^ ( Figure 1). Interestingly, each of these approaches is based on the manipulation of a single gene, but they have enormous potential to not only reduce Pi or N applications but also have a profound environmental impact. In the following paragraphs, these approaches and their implications are discussed.

***Alanine aminotransferase.*** Alanine aminotransferase (AlaAT) plays an important role in carbon fixation and N metabolism because it catalyzes the reversible reaction between pyruvate and glutamate to produce alanine and oxoglutarate ^43^. The potential effectiveness of this approach relies on the facts that amino acids act as signals controlling N uptake and that alanine is the only amino acid whose biosynthesis is not inhibited by N deficiency ^43^. The development of the AlaAT technology started with the expression of barley AlaAT in canola using the *btg26* root-specific promoter, which resulted in the production of increased biomass under low-N conditions ^26^. Field evaluations showed that *btg26:*AlaAT canola plants exhibited a 42% increase in seed yield under suboptimal N fertilization (56 kg ha ^-1^). This yield increase correlated with lower levels of glutamine and glutamate in the shoot, increased N influx and higher levels of alanine in roots, and up-regulation of high-affinity N-transporters. The overexpression of barley AlaAT in rice, which was also driven using a root-specific promoter ( *OsANT1*), also resulted in increased biomass and grain yield ^44^. Recently, similar results have been reported for sugarcane ^45^.

Interestingly, the *btg26* promoter is expressed mainly in the cortex and lateral roots of transgenic plants ^26^, which are fundamental for the uptake and loading of nutrients into the vascular system. It will be interesting to determine how robust the AlaAT-overexpressing phenotype is under different stress conditions and soil types.

***Phosphorus-starvation tolerance 1.*** Because of the low mobility of Pi in the soil ^7^, a well-developed and highly branched root system is a determinant for soil exploration and Pi uptake in soils with a low availability of this nutrient ^46^. However, modern breeding programs have focused on developing high-yield crops by primarily selecting the above-ground phenotype and applying full fertilization during breeding processes, which probably selects against root traits that are important for nutrient uptake efficiency. The QTL *Pup1* (Phosphorus Uptake1), which is responsible for low-Pi tolerance, was identified in a cross between a low-Pi-tolerant rice landrace with a low-Pi-intolerant modern rice variety ^41^. *Pup1* was found to contribute to enhanced Pi uptake and grain yield by 170% and 250%, respectively, in low-Pi soils in the modern variety ^47^. Recently, it was found that the *PSTOL1* gene, which encodes a protein kinase, is responsible for the effect of the *Pup1* QTL on Pi uptake and assimilation ^41^. The overexpression of *PSTOL1* under a constitutive promoter (CaMV35S) in two types of modern rice varieties (one indica and one japonica) that naturally lack the gene resulted in an increase of over 60% in grain yield in low-Pi soils ^41^.

Interestingly, the low-P tolerance phenotype conferred by PSTOL1 correlated with a more robust root system, as transgenic plants produced almost five times more root biomass than did the non-transgenic plants. A global expression analysis of the PSTOL1-overexpressing plants revealed a set of up-regulated genes that are related to root growth and stress responses, including a putative peptide transporter ^41^. Because peptide transporters are included in the set of N-transporters (PTR/NRT1) in plants ^48^, it would be interesting to determine whether *PSTOL1*-overexpression could also improve N uptake efficiency. Optimizing the cell-specific and regulated expression of *PSTOL1* will probably have an even higher impact on grain yield.

***The phosphite oxidoreductase/phosphite system.*** The high reactivity of Pi with soil components and the constant competition of microorganisms and weeds with cultivated plants make agriculture highly dependent on P-fertilizers and herbicides. Recently, a phosphite oxidoreductase (PTXD) from *Pseudomonas stutzeri* was used to propose a re-design of the currently used agricultural systems ^42^. PTXD oxidizes Phi using NAD ^+^ as a cofactor and yields Pi and NADH as products ^49^. The expression of PTXD in *Arabidopsis* and tobacco produced transgenic plants that are capable of using Phi as a sole P source.

The importance of this approach relies on the fact that Phi has distinct chemical and biochemical properties compared with Pi, including higher solubility and lower reactivity with soil components ^50^; in addition, plants and most microorganisms are unable to metabolize Phi as a P source ^51– 53^. Therefore, the system makes the PTXD-transgenic plants more competitive over other plants, including weeds, in low-Pi soils that are fertilized with Phi. PTXD-transgenic lines required 30–50% less P to achieve optimal productivity when they were fertilized with Phi instead of Pi ^42^ and reduced the requirement of herbicides because of the poor growth of weeds in soils fertilized with Phi.

This technology is still in its infancy, and several questions need to be addressed before its real potential is uncovered. The promising results obtained under greenhouse and field conditions suggest that a potential reduction in production costs and energy consumption could be achieved by replacing the independent application of fertilizer and herbicides with a single treatment and by reducing the cost of additional herbicides. It is important to consider that the PTXD/Phi system has the highest potential for use in acidic and alkaline soils that have very low-Pi availability and that, in spite of having appropriate climatic conditions and water availability to sustain high crop productivity, it has been used only as grassland for cattle. These areas are predominant in Brazil, China, Australia, India, and Russia, comprising over 350 million hectares that could be converted into highly productive cropping areas if the PTXD/Phi technology was incorporated into genotypes adapted to acidic and alkaline soils.

This technology provides sustainable management of P, and thus has the potential to prolong the lifetime of phosphate rock reserves and to reduce the environmental impact of eutrophication of lakes, seas, and oceans. Additionally, a paper published recently suggested that on the moon or other planets that lack oxygen in the atmosphere, on which P accumulates primarily as schreibersite mineral and that are rich in Phi, the PTXD/Phi technology could be an interesting alternative for establishing agriculture ^54^.

## Element toxicities that limit crop productivity

There are other nutritionally related stresses that have an important impact on plant yield that deserve special attention, which we only briefly mention because they are outside the main scope of this review, namely aluminum (Al) and boron (B) toxicity. Al toxicity is a major constraint for plant yield on acidic soils, which comprise between 40 and 50% of the world’s potentially arable lands. At pH values below 5, Al ^3+^ ions are dissolved from soil minerals and are highly toxic to plants, impairing root growth and function. Two main classes of Al resistance mechanisms have been reported: Al exclusion mechanisms, which prevent Al from entering the root apex, and Al tolerance mechanisms, in which Al enters the plant but is sequestered into the vacuole and detoxified. Since the root apex is the main site of Al toxicity, the most well-characterized exclusion mechanism involves the regulated release of organic acids (OAs) by the root tip, which chelate Al ^3+^ ions forming non-toxic compounds that do not enter the root tip cells. Members of the Al-activated Malate Transporter (ALMT) family of anion channel transporters and the Multidrug and Toxic compound Extrusion (MATE) family of OA/H ^+^ antiport transporters are responsible for plasma membrane malate and citrate efflux, respectively, from root cells into the rhizosphere in response to the presence of toxic concentrations of Al ^3+^ ions (for a review see 55). Several attempts have been made to show that overexpression of MATE and ALMT genes leads to enhanced Al tolerance ^56^. However, the effectiveness of OA efflux transporters to confer an enhanced Al ^3+^ tolerance remains to be demonstrated under field conditions and also to be agronomically relevant.

B is an essential micronutrient required for several physiological and developmental processes in plants, including meristem development, but that can also be present in toxic levels in the soil. Typical B toxicity symptoms include necrosis of marginal leaves and the inhibition of root growth (for a review see 57). It has been revealed over the last 10 years that plants have B transporters that maintain B homeostasis. B tolerance loci have been identified in high B-tolerant barley and wheat genotypes, which encode B exporters to reduce B concentrations in roots and to alter cellular distribution of B in shoots that are absent in susceptible lines. In barley, tolerance to toxic levels of B is associated with four tandem copies of *Bot1* (encoding a B efflux transporter), which is highly expressed in the tolerant landraces ^58^, whereas B tolerance in wheat is associated with a B transporter-like gene ( *Bot-B5b*) that has high root expression levels in tolerant genotypes as compared to susceptible lines ^59^. The finding that high expression of B exporters reduces B concentration in the plant, or that a decreased expression of the transporters that facilitate B uptake could lead to tolerance to toxic B levels, opens up the possibility of using transgenic approaches or genome editing technologies to improve the yield of different crops in soils containing toxic levels of B.

## Engineering tolerance to drought, salinity, and high temperatures

Drought, saline soils, and extreme temperature are abiotic stresses that adversely affect the growth and productivity of most crops. Drought is the most aggressive form of osmotic stress and limits crop yield in approximately 50% of the total cultivated area worldwide ^60^. Table 1 shows the numerous efforts to engineer crops for drought tolerance. Plants have evolved adaptive mechanisms to cope with abiotic stresses by remodeling morphological and physiological processes, mainly by altering their metabolism to reduce transpiration and promote osmotic adjustment through the interaction of multiple signaling pathways. Adaptive mechanisms that allow plants to cope with drought, salinity, and high temperatures include the production and accumulation of osmoprotectants, molecular chaperones, and antioxidants. Osmoprotectants are metabolites that protect cells by maintaining their water potential and by stabilizing membranes and scavenging reactive oxygen species (ROS) ^61^. Heat shock proteins (HSPs) and late embryogenesis abundant (LEA) proteins also play crucial roles during seed desiccation and water stress by preventing protein denaturation and aggregation ^62^. Additionally, several genes that encode TFs have been identified as key elements that possess the potential to improve crop performance under different abiotic stresses ( Table 1). This section discusses the most promising approaches to engineering crops with enhanced tolerance to drought stress, extreme temperatures, and soil salinity.

**Table 1.** Transgenic approaches to improve tolerance to drought and other abiotic stresses. Numerous genes have been shown to improve drought-tolerance in transgenic crops. In addition, some of these approaches have improved productivity and tolerance to other abiotic stresses, such as cold, heat, and high salinity. The gene source and the type of expression system—constitutive (C), inducible (I) or tissue specific (TS)—are indicated in each case. Positive and negative phenotypic alterations are also specified when data are available (GR, growth retardation; IB, increase biomass; PE, pleiotropic effect; SA, sensitivity to ABA; SOx, increased sensitivity to oxidative stress). Gene sources: *Arabidopsis thaliana* (At), *Arthrobacter globiformis* (Ag), *Bacillus subtilis* (Bs), *Cynodon dactylon* x *C. transvaalensis* (Cdt), *Escherichia coli* (Ec), *Glycine max* (Gm), *Gossypium arboreum* (Ga), *Hordeum vulgare* (Hv), *Macrotyloma uniflorum* (Mu), *Malus domestica* (Md), *Medicago truncatula* (Mt), *Nicotiana tabacum* (Nt), *Oryza sativa* (Os), *Pisum sativum* (Ps), *Solanum habrochaites* (Sh), *Solanum lycopersicum* (Sl), *Solanum tuberosum* (St), *Thellungiella halophile* (Th), *Triticum aestivum* (Ta), *Vigna aconitifolia* (Va). ND, not data.

Functional category	Gene family	Gene	Transformed crop	Tolerance				Genetic source	Expression	Productivity	Field evaluation	Phenotypic alterations	Reference
				Salt	Cold	Heat	Others						
**Transcription** **factor**	**AP2/ERF**	DREB1A	Wheat					At	I				109
		CBF3/ DREB1A	Rice	✔				At	C				110
		OsDREB1	Rice	✔	✔			Os	C			GR	111
		AtDREB1A	Peanut	✔				At	I				112
		OsDREB2A	Rice	✔				Os	I				113
		TaDREB2	Wheat					Ta	I				77
		TaDREB2	Barley		✔			Ta	C				77
		TaDREB3	Wheat					Ta	I				77
		TaDREB3	Barley		✔			Ta	C			GR	77
		HvCBF4	Rice	✔	✔			Hv	C				114
		TaERF3	Wheat	✔				Ta	C				115
		OsERF4a	Rice					Os	C, I			SA	116
		SlERF5	Tomato	✔				Sl	C				117
		AP37	Rice	✔	✔			Os	C	✔	✔		118
		AP59	Rice	✔				Os	C		✔		118
		TSRF1	Rice				✔	Sl	C				119
		JERF1	Rice					Sl	C				120
	**bZIP**	SlAREB1	Tomato	✔				Sl	C				121
		AtAREB1 (active form)	Soybean					At	C				122
		ABF3	Rice					At	C				110
		GmbZIP1	Wheat					Gm	C				123
		OsbZIP16	Rice					Os	C			SA	124
		OsbZIP23	Rice	✔				Os	C			SA	125
		OsbZIP46 (active form)	Rice				✔	Os	C			SA	126
		OsbZIP72	Rice					Os	C			SA	127
	**NAC**	SNAC1	Rice	✔				Os	C		✔	SA	71
		SNAC1	Wheat	✔				Os	C	✔		SA	128
		SNAC1	Cotton	✔				Os	C	✔			129
		MuNAC4	Peanut					Mu	C				130
		OsNAC5	Rice					Os	C, TS	✔	✔		131
		OsNAC6	Rice	✔			✔	Os	C			GR	132
		OsNAC9	Rice					Os	C, TS	✔	✔		133
		OsNAC10	Rice	✔	✔			Os	C, TS	✔	✔		75
		ONAC045	Rice	✔				Os	C				134
		TaNAC69	Wheat	✔				Ta	I	✔			135
	**NF-Y**	ZmNF-YB2	Maize					Zm	C	✔	✔		72
		CdtNF-YC1	Rice	✔				Cdt	C			SA	136
	**MYB**	StMYB1R-1	Potato					St	C				137
		OsMYB2	Rice	✔	✔			Os	C			SA	138
		OsMYB48-1	Rice	✔				Os	C			SA	139
		MdoMYB121	Tomato and apple	✔	✔			Md	C				140
		TaPIMP1	Wheat				✔	Ta	C				141
	**WRKY**	OsWRKY11	Rice			✔		Os	I				142
		OsWRKY30	Rice					Os	C				143
	**Zinc finger**	ZFP252	Rice	✔				Os	C				144
		ZAT10	Rice					ND	C, I	✔	✔		145
	**Combination** **of different** **TFs**	AtDREB2A, AtHB7 and AtABF3	Peanut	✔			✔	At	C				146
	**Other** **transcription** **factors**	OsiSAP8	Rice	✔	✔			Os	C				147
		WXP1	Alfalfa					Mt	C				148
**Protein** **kinases**	**MAPKs**	OsMAPK5	Rice	✔	✔			Os	C				149
		NPK1	Maize					Nt	C				150
		DSM1	Rice					Os	C				151
	**CIPK**	MdCIPK6L	Tomato	✔	✔			Md	C				152
		OsCIPK12	Rice					Os	C				153
	**CDPK**	OsCDPK1	Rice					Os	C				154
		OsCDPK7	Rice	✔	✔			Os	C				155
		OsCPK4	Rice	✔				Os	C				156
		OsCPK9	Rice					Os	C			SA	157
	**Other protein** **kinases**	OsSIK1	Rice	✔				Os	C				158
**Metabolism of** **hormones**	**ABA**	DSM2	Rice				✔	Os	C				159
		LOS5	Rice					ND	C, I	✔	✔		145
		AtLOS5	Cotton					At	C	✔			160
		LOS5	Soybean					At	C	✔	✔		161
		LOS5	Maize					At	C				162
	**Citokinin**	IPT	Peanut					ND	I		✔		163
		IPT	Rice					ND	I				164
		IPT	Cotton					ND	I				165
	**Auxin**	OsPIN3t	Rice					Os	C				166
**Osmolytes**	**Trehalose**	OsTPS1	Rice	✔	✔			Os	C				161
		otsA and otsB	Rice	✔	✔			Ec	I, TS				68
		TPS and TPP	Rice	✔	✔			Ec	C				69
	**Proline**	P5CS	Wheat					Va	I				167
	**Mannitol**	mtlD	Wheat	✔				Ec	C				168
	**Glycine** **betaine**	betA	Maize					Ec	ND	✔			169
		codA	Potato	✔			✔	Ag	I				170
		codA	Tomato	✔				Ag	C				171
**Responsive** **stress** **proteins**	**LEA** **proteins**	OsLEA3-1	Rice					Os	C, I	✔			63
		OsLEA3-2	Rice	✔				Os	C				172
		HVA1	Rice	✔				Hv	C	✔			65
		HVA1	Wheat					Hv	C				173
		HVA1	Wheat					Hv	C	✔	✔		64
	**Dehydrin**	TAS14	Tomato	✔				Sl	C				174
		ShDHN	Tomato		✔			Sh	C				175
	**HSP**	GHSP26	Cotton					Ga	C				66
	**Cold** **shock** **proteins**	CspA and CspB	Maize					Ec, Bs	C	✔	✔		67
		CspA or CspB	Rice		✔	✔		Ec, Bs	C	✔			67
**Transporters**		NHX1	Rice					ND	C, I	✔	✔		145
		betA and TsVP	Maize					Ec, Th	C	✔			176
		AVP1	Cotton	✔				At	C	✔	✔		177
**Antioxidant** **enzymes/** **compounds**		OsSRO1c	Rice					Os	C			SOx	178
		MnSOD	Rice					Ps	I				179

### Manipulation of LEA and HSP genes

HSPs and LEA proteins from plants have been clearly shown to be involved in abiotic stress responses; however, as shown in Table 1, only limited attempts have been made to use the genes that encode these proteins to engineer abiotic stress tolerance in crops. Nevertheless, there are some examples that show the potential of overexpressing LEA proteins in vegetative tissues. For instance, the constitutive expression of *OsLEA3-1* in rice ^63^ and *HvLEA1* in wheat ^64^ and rice ^65^ resulted in improved yields under drought stress without impairing yield under control conditions. Similarly, overexpressing *GHSP26* resulted in improved drought and osmotic stress tolerance in cotton plants ^66^. However, although transgenic plants that constitutively express LEA- and HSP-encoding genes have shown improved abiotic stress tolerance under both *in vitro* and greenhouse conditions, their efficacy under field conditions remains to be demonstrated ( Table 1). Interestingly, the best results were obtained in transgenic plants expressing the *cold shock protein A (CspA)* and *CspB* genes from *Escherichia coli* and *Bacillus subtilis,* respectively. These genes encode RNA-binding proteins with chaperone activity that confer drought tolerance in maize and rice under field conditions ^67^. In fact, *CspB*-expressing maize is the first genetically modified (GM) crop with enhanced water use efficiency that has been deregulated for commercial use in the USA ^5^.

The multiple pathways involved in plant adaptations to osmotic and water stress and the complexity of their interactions can explain, to some extent, the limited success under field conditions of manipulating individual genes encoding chaperones or enzymes involved in the synthesis of osmoprotectants ^68, 69^. To develop crops with higher yields under drought, it will most likely be necessary to engineer metabolic pathways through the simultaneous manipulation of multiple critical genes. In addition, it would be interesting to explore the mechanisms that regulate desiccation tolerance in seeds to obtain new insights into the adaptive stress response pathways and to identify new candidate genes for crop improvement.

### Manipulation of regulatory genes

Manipulating proteins that regulate gene expression or the signal transduction of multiple metabolic pathways involved in abiotic stresses has proven to be useful for improving the stress tolerance of crops ( Table 1). TFs that belong to the Dehydration-Responsive Element-Binding/C-repeat Binding Factor (DREBs/CBF) ^70^, NAM-ATAF and CUC (NAC) ^71^, and Nuclear Factor Y (NF-Y) ^72^ families have been used to develop transgenic plants and study their performance under stress conditions. The expression of some of these TFs under drought-inducible or root-specific promoters has resulted in improved tolerance to drought, salinity, and temperature stress and a higher yield under water-limited conditions in rice ^73– 76^, wheat ^77^, canola ^78^, and maize ^72^.

## Genomic resources for breeding crops with enhanced abiotic stress tolerance

As observed for N and P improvement, hundreds of QTLs related to drought and heat tolerance traits have been identified. However, only a few of them have been implemented in appropriate breeding programs for improving crop abiotic stress tolerance. Efforts have been made to improve drought tolerance in rice by using marker-assisted (MAS) breeding ^79^ to identify and characterize the Deeper Rooting 1 (DRO1) QTL that controls the root growth angle ^80^. Higher expression of DRO1 causes a more vertical root growth. Breeding DRO1 into a shallow-rooting rice line enables these plants to avoid drought by increasing the depth of their roots, resulting in a higher grain yield ^80^. The DRO1 gene is the first drought tolerance QTL that was cloned, and its beneficial effects on plant growth further confirmed that the root system architecture plays a crucial role in abiotic stress tolerance. Interestingly, *DRO1* has no homology to known proteins, which suggests that cloning genes associated with QTLs could provide completely novel genes for plant breeding. This example shows that a considerable improvement in drought tolerance can be achieved by altering root growth patterns and opens up the possibility of introducing DRO1 in shallow-rooting crops other than rice through the use of genetic engineering ( Figure 1).

The phytohormone abscisic acid (ABA) regulates numerous processes in plants including seed dormancy and the plant responses to low water availability. ABA is perceived by soluble PYR/PYL/RCAR (pyrabactin resistance1/PYR1-like/regulatory component of ABA receptor) receptors that belong to the START superfamily of ligand-binding proteins (for a review see 81). It has been shown that constitutive overexpression of ABA receptors improves drought tolerance; however, it negatively affects yield under non-stress conditions ^82^. This suggests that the precise regulation of the activity of individual or multiple receptors will be required to achieve enhanced drought tolerance without a yield penalty. A novel alternative to actively control tolerance to abiotic stress is the use of chemicals that can activate or repress the receptors that sense the stress or the signaling pathways activated by hormones that mediate the corresponding stress responses. Recently it was shown that it is feasible for the case of drought tolerance. Drought tolerance in *Arabidopsis* was achieved using an engineered PYR1 ABA-receptor that can be activated by an existing non-herbicidal agrochemical that is not a natural inducer of ABA responses. This example opens up a new avenue of crop improvement to regulate abiotic or biotic stress responses at the beginning of, or prior to, the presence of the stress in a timely and quantitative manner by the application of a non-toxic compound, reducing potential yield reductions ^83^.

## The hidden enemy in the soil: mechanical impedance

Among the different types of soil physical degradation, soil compaction is considered one of the most serious problems in agricultural fields because it directly alters the soil structure and modifies intrinsic soil properties, such as porosity, aeration, water potential, and soil strength ^84^. Soil compaction increases soil impedance and thereby affects crop yield by decreasing the capacity of the root system to explore new soil horizons and absorb water and essential nutrients to sustain active growth and development. Several studies have shown that continuous mechanical impedance affects root system architecture by altering root diameter, total root length, and lateral root initiation ^85– 87^. Despite the increasing importance of soil compaction resulting from the mechanization of agriculture, this abiotic stress is the least studied to date.

**Figure 2.**
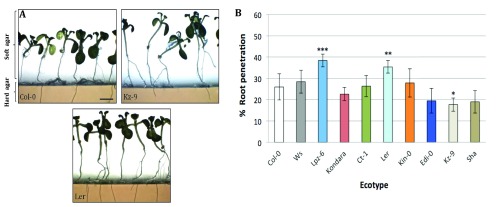
Natural variation of *Arabidopsis* ecotypes in root penetration ability. **A**) Col-0, Kz-9 and Ler *Arabidopsis* ecotypes show a wide variation in penetrating hard agar layers. Screening test was carried out using a double-phase agar system, which mimics soil compaction condition. **B**) Quantitative analysis of the root penetration ability expressed as the root penetration percentage (%) in reference to that of Col-0, showed by nine different *Arabidopsis* ecotypes. (*) indicates statistically significant differences: * *P*<0.05, ** *P*<0.01, and *** *P*<0.001 level; n=120 seedlings per ecotype (one-way ANOVA).

Studying the genetic diversity of root penetration ability could permit the identification and characterization of genes that allow roots to penetrate soils with high impedance. Genotypic variation in root penetration ability has been found in soybean ^88^, rice ^89^, and wheat ^90^. Our group has found that, among *Arabidopsis* ecotypes, there is wide variation in the capacity of the root system to penetrate substrates with high mechanical impedance ( Figure 2). At the molecular level, some studies have attempted to elucidate the detailed mechanosensing and mechanotransduction processes in roots by studying early signaling events during physical stimuli and the role of putative mechanoreceptors ^91– 93^. Although important advances have been made in this field, the precise mechanisms and specific root traits that enable roots to penetrate into hard soils remain largely unknown. Several interesting questions still need to be answered with regard to root penetration. Why can some plant species more efficiently penetrate compact soil layers? Which genes are involved in the adaptive root traits that permit some plant species or genotypes to effectively cope with soil compaction problems? And what hormonal changes occur when a plant encounters a below-ground obstacle?

The use of image analysis techniques based on transparent substrates and 3D imaging using X-ray and neutron tomography technologies or fluorescent and luminescent proteins in conjunction with specifically designed devices should improve our understanding of how roots respond to high mechanical impedance with much better resolution, compared with that previously possible at the macroscopic level ^94– 96^.

The plant cell wall consists primarily of polysaccharides that can be broadly classified as cellulose, cellulose-binding hemicelluloses, pectins, and lignins, which confer mechanical stability and allow adequate cell expansion through the regulation of turgor pressure generated inside plant cells ^97, 98^. Enzymes, such as endoglucanases, xyloglucan-endotransglycoxylases and expansins, play crucial roles in mediating the rearrangement of the cell wall structure. Modulating the expression of the genes involved in the synthesis and remodeling of cell wall components could allow the modification of root mechanical properties to produce stronger root systems that have a better capacity to penetrate compacted soils ( Figure 1). In *Arabidopsis*, specific TFs, such as MYB58 and MYB63, have been found to activate lignin biosynthetic genes during secondary wall formation ^99^. Therefore, the overexpression of these TFs under root-specific or stress-inducible promoters could result in plant roots that have strengthened cell walls with enhanced tolerance of mechanical restriction ^100^ ( Figure 1).

It is essential to consider root responses to soil compaction in current and future breeding programs. In conjunction with genetic engineering and genome editing technologies, this approach will accelerate the development of crop varieties with enhanced performance in soils degraded by compaction.

## Concluding remarks

As discussed above, engineering for tolerance to abiotic stress by manipulating key genes and using multiple tools has allowed the generation of crop plants that are tolerant to drought, extreme temperatures, and salinity, or that have a higher nutrient uptake and use efficiency. A remarkable contribution has resulted from studies with tolerant crop varieties to certain stresses instead of using model genotypes, such as the case of the PSTOL1, suggesting that we must encourage the use of tolerant genotypes in our research.

The pursuit of master regulators that control abiotic stress and determination of the best way to modulate their expression has been the most important challenge in engineering plant genetics to enhance abiotic stress tolerance. However, rapid advances in genomic technologies for the characterization of QTLs and performing genome-wide association studies ^101^ should facilitate the identification of novel genes for engineering abiotic stress tolerance in crops. The use of systems biology that integrates “omics” data ^102^ and generates mathematical models to achieve a more complete view of the interactions between plant responses to abiotic stress should also facilitate the design of effective strategies to engineer plants with enhanced performance under harsh conditions.

Epigenetic processes, such as DNA methylation, histone modifications, generation of small RNAs (sRNA), and transposable element activity, play essential roles in modulating gene activity in response to environmental stimuli ^103, 104^. Indeed, it has been shown that drought adaptive-responses in plants can be transgenerationally transmitted through the action of these processes on specific genes ^105^. Moreover, epigenetic processes are also involved in the switch from C3 to CAM photosynthesis and contribute to adaptation to salt stress in the halophyte *Mesembryanthemum crystallinum* ^106^. In wheat, the use of the methylation inhibitor 5-azacytidine resulted in increased tolerance to salt stress at the seedling stage ^107^. Therefore, understanding the epigenetic mechanisms that control gene expression in response to environmental cues could also become an important avenue for developing improved crops ( Figure 1). However, more information is needed to clarify the complex interaction between abiotic stress responses and epigenetic changes and to identify potential stress-responsive epigenetic modifiers.

We believe that the most exciting transgenic approaches for producing plant varieties and hybrids that are much less dependent on the application of agrochemicals, including fertilizers and pesticides, have yet to be discovered. The engineering of crops for harsh environments is evolving and will rapidly incorporate new breeding technologies, including genome editing, which has already produced its first commercial product (herbicide-resistant canola). The development of effective approaches for specifically and visibly monitoring certain environmental stresses, such as P deficiency, and timely indicating the degree of the stress is also emerging and providing additional tools for improving crops ^108^. Furthermore, the possibility of activating or repressing the expression of specific genes by introducing site-specific epigenetic changes, such as DNA methylation or histone modifications using a modified version of the CRISPR/Cas9 system ^99^, will drastically modify how agriculture is developed by creating an integral, effective, and sustainable global agriculture. However, translating these approaches from the laboratory or the greenhouse to the field remains challenging. In our opinion, more interdisciplinary research and the active involvement of breeders and agronomists in project planning is necessary to better define project goals and align the interests of researchers with that of crop producers.
