# Supplementary material for: Engineering food crops to grow in harsh environments
Source: F1000Res. 2015 Sep 2;4(F1000 Faculty Rev):651. [Version 1] doi: 10.12688/f1000research.6538.1 (PMC4560252; doi:10.12688/f1000research.6538.1)
Supplement: Peer review history

# Peer review: Referee response for version 1

**DOI:** 10.5256/f1000research.7021.r10205

**Published:** 2015-09-02

**Authors:** Sigrid Heuer

**Affiliations:** 1 Australian Centre for Plant Functional Genomics (ACPFG), Glen Osmond, SA 5064, Australia

I have read this submission. I believe that I have an appropriate level of expertise to confirm that it is of an acceptable scientific standard.

# Peer review: Referee response for version 1

**DOI:** 10.5256/f1000research.7021.r10204

**Published:** 2015-09-02

**Authors:** Guohua Xu

**Affiliations:** 1 State Key Laboratory of Crop Genetics and Germplasm Enhancement, College of Resources and Environmental Sciences, Nanjing Agricultural University, Nanjing, 210095, China

I have read this submission. I believe that I have an appropriate level of expertise to confirm that it is of an acceptable scientific standard.
